# StoneMod: a database for kidney stone modulatory proteins with experimental evidence

**DOI:** 10.1038/s41598-020-71730-3

**Published:** 2020-09-15

**Authors:** Supatcha Sassanarakkit, Paleerath Peerapen, Visith Thongboonkerd

**Affiliations:** Medical Proteomics Unit, Office for Research and Development, Faculty of Medicine Siriraj Hospital, Mahidol University, 6th Floor - SiMR Building, 2 Wanglang Road, Bangkoknoi, Bangkok, 10700 Thailand

**Keywords:** Bioinorganic chemistry, Proteins, Protein databases, Renal calculi, Urology

## Abstract

Better understanding of molecular mechanisms for kidney stone formation is required to improve management of kidney stone disease with better therapeutic outcome. Recent kidney stone research has indicated critical roles of a group of proteins, namely ‘*stone modulators*’, in promotion or inhibition of the stone formation. Nevertheless, such information is currently dispersed and difficult to obtain. Herein, we present the kidney stone modulator database (StoneMod), which is a curated resource by obtaining necessary information of such stone modulatory proteins, which can act as stone promoters or inhibitors, with experimental evidence from previously published studies. Currently, the StoneMod database contains 10, 16, 13, 8 modulatory proteins that affect calcium oxalate crystallization, crystal growth, crystal aggregation, and crystal adhesion on renal tubular cells, respectively. Informative details of each modulatory protein and PubMed links to the published articles are provided. Additionally, hyperlinks to other protein/gene databases (e.g., UniProtKB, Swiss-Prot, Human Protein Atlas, PeptideAtlas, and Ensembl) are made available for the users to obtain additional in-depth information of each protein. Moreover, this database provides a user-friendly web interface, in which the users can freely access to the information and/or submit their data to deposit or update. Database URL**:** https://www.stonemod.org.

## Introduction

**Figure 1:**
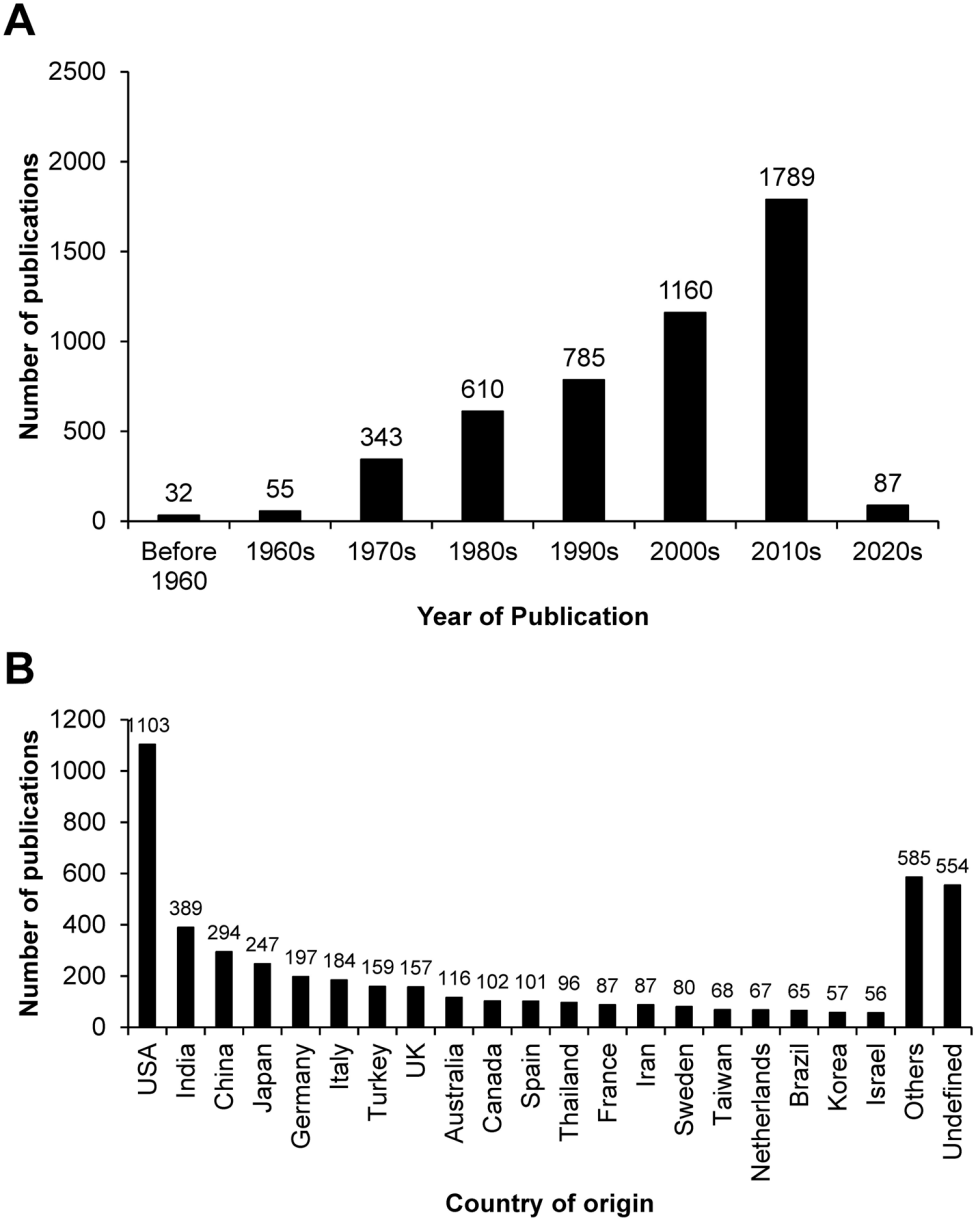
Number of the published articles related to the investigations of kidney stone modulation based on year of publication (**A**) and country of origin (**B**). (Others = 58 countries other than what have shown; Undefined = the affiliations were not provided in the PubMed database).

Kidney stone disease is a common health problem worldwide. Although precise mechanisms of kidney stone formation remain unclear, two main hypotheses involving intratubular supersaturation of causative ions (e.g., calcium and oxalate ions) and interstitial deposition of the causative crystals (i.e., Randall’s plague model) are widely accepted^1,2^. Nonetheless, the incidence/prevalence of this disease has been increasing^3^, reflecting ineffective prevention and poor understanding of the disease pathogenesis. Therefore, stone research during the past few decades has attempted to address mechanisms of kidney stone formation at tissue, cellular, subcellular and molecular levels^4,5^. One aspect of such research that draws lots of attention from many researchers is the study on crystal modulation with the ultimate goals to better understand the processes of crystallization, crystal growth, crystal aggregation, and crystal adhesion to renal tubular cells^1,2^, which are essential for kidney stone formation, and to define strategy to inhibit these processes^4,5^. The study on kidney stone modulation has been extensively investigated by numerous groups from > 70 countries and various regions and there have been > 4,800 PubMed articles published from such investigations since 1900s (Fig. 1).

In the urine and kidney tissue, there are a group of molecules, including proteins, which can modulate kidney stone formation by either promoting or inhibiting each step of the stone formation processes. For example, urinary trefoil factor 1 (TFF1) can inhibit calcium oxalate crystal growth and aggregation^6,7^, whereas bikunin (urinary trypsin inhibitor or α-1-microglobulin) can inhibit calcium oxalate crystallization, growth, and aggregation^8,9^. However, some stone modulatory proteins have shown ambiguous effects on stone modulation as in the case of Tamm-Horsfall protein (uromodulin), which promotes calcium oxalate crystal aggregation, but on the other hand, inhibits crystal growth^10,11^. Unfortunately, these lines of references are dispersed and such contradictory results may easily generate confusion despite enormous efforts in kidney stone research. It is thus essential to generate a resource or database for kidney stone modulators that allows researchers to rapidly and accurately obtain precise information of the existing modulators and their effects on kidney stone formation.

We present herein the kidney stone modulator database (StoneMod), which curates and catalogues all the kidney stone modulators with experimental evidence from previously published studies. Because calcium oxalate is the most common type of kidney stones and occupies > 77% of 111,196 stones analyzed^12^ and assays for investigating calcium oxalate kidney stone formation processes were well-established, this version of the database focuses on modulators of calcium oxalate crystallization, crystal growth, aggregation, and adhesion to renal tubular cells.

## Results and discussion

### Overview of the StoneMod database

**Figure 2:**
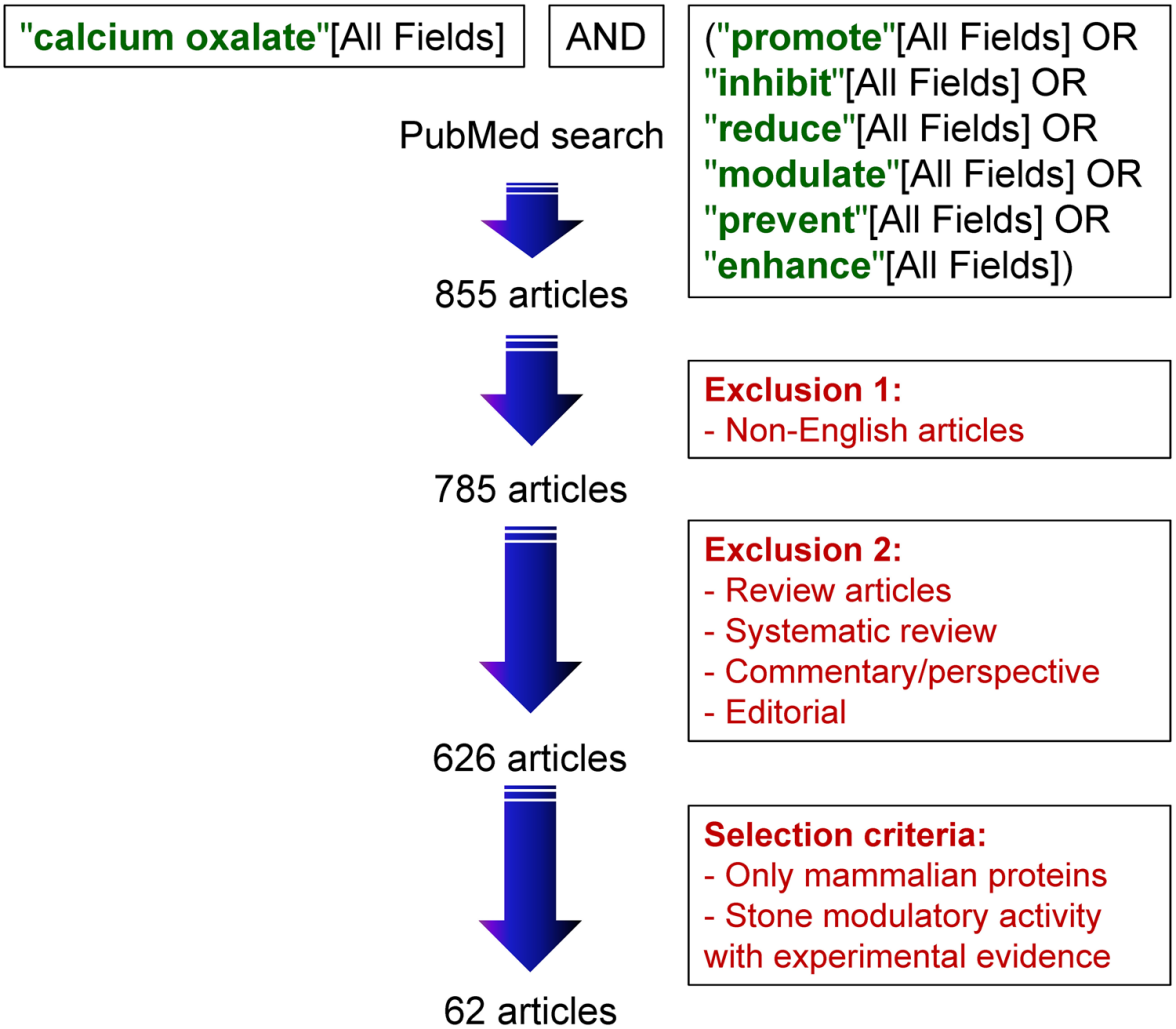
Schematic workflow of data collection and curation to generate the StoneMod database.

The aim of our present work was to build a database that integrates all relevant information of kidney stone modulators with experimental evidence. The StoneMod database provides a collection of modulatory proteins that either promote or inhibit individual steps of kidney stone formation. Using the predefined criteria for inclusion/exclusion (Fig. 2) (see more details in “Materials and Methods”), the StoneMod database currently contains 10, 16, 13, 8 modulatory proteins that affect calcium oxalate crystallization, crystal growth, crystal aggregation, and crystal adhesion on renal tubular cells, respectively (Table 1). All of these data were retrieved from 62 published studies, involving urine, serum, cellular secretome, and kidney tissue samples (Supplementary Table S1). Informative details of each modulatory protein and PubMed links to the published articles are provided. Additionally, hyperlinks to other protein/gene databases (i.e., UniProtKB, Swiss-Prot, Human Protein Atlas, PeptideAtlas, and Ensembl) are made available for the users to obtain additional in-depth information of each protein.

**Table 1:** Summary of the entries included in the StoneMod database.

Modulatory function	No. of proteins	No. of studies
**Crystallization**	**10**	**23**
Inhibitor	7	10
Promoter	–	–
Contradictory	3	13
**Crystal growth**	**16**	**32**
Inhibitor	13	29
Promoter	2	1
Contradictory	1	2
**Crystal aggregation**	**13**	**24**
Inhibitor	11	13
Promoter	1	1
Contradictory	1	10
**Crystal adhesion**	**8**	**15**
Inhibitor	6	9
Promoter	–	-
Contradictory	2	6

### The tabbed document interface

**Figure 3:**
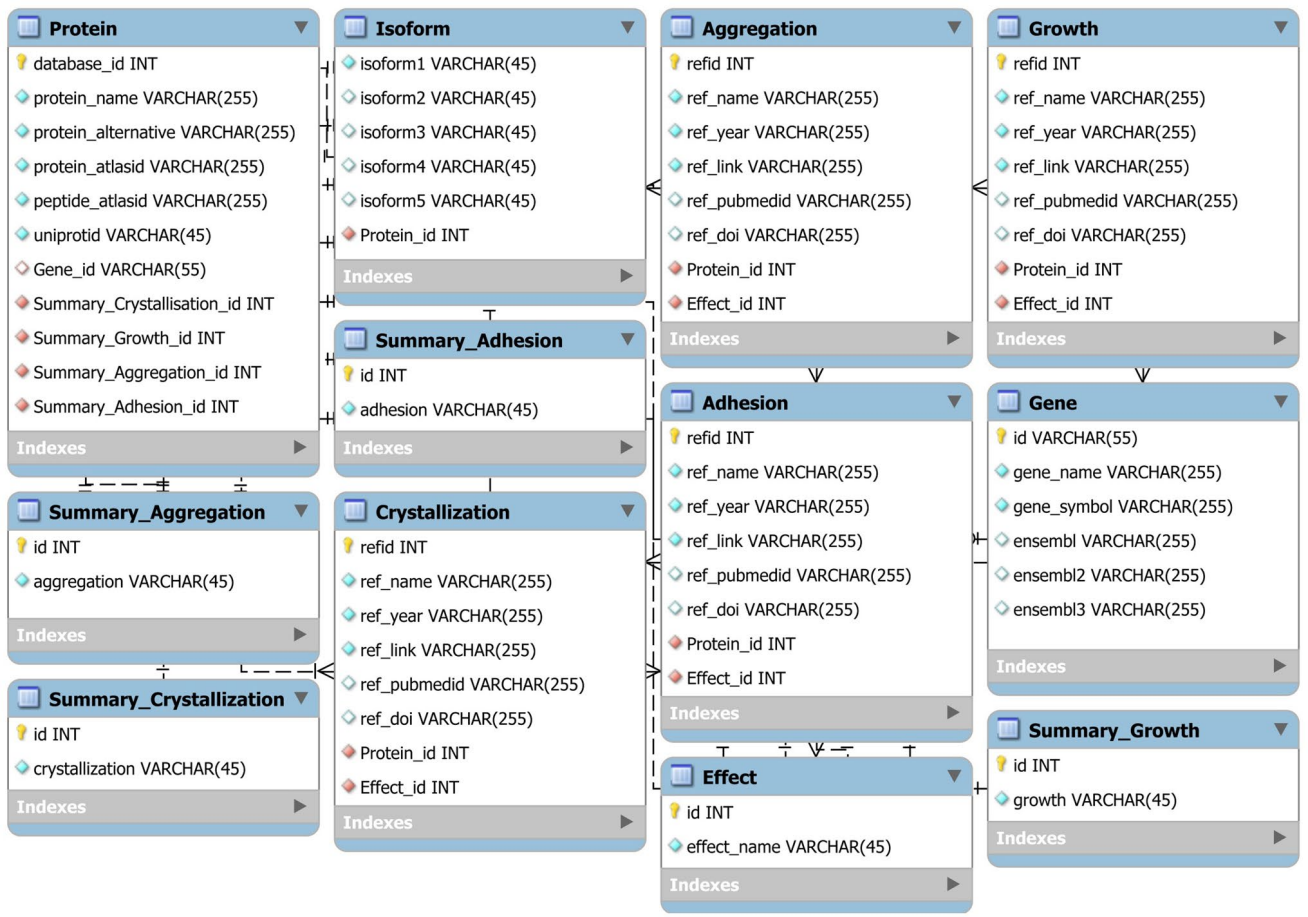
MySQL schema used for generation of the StoneMod database. The main relational database consists of twelve tables, representing all related parameters used for the database construction.

**Figure 4:**
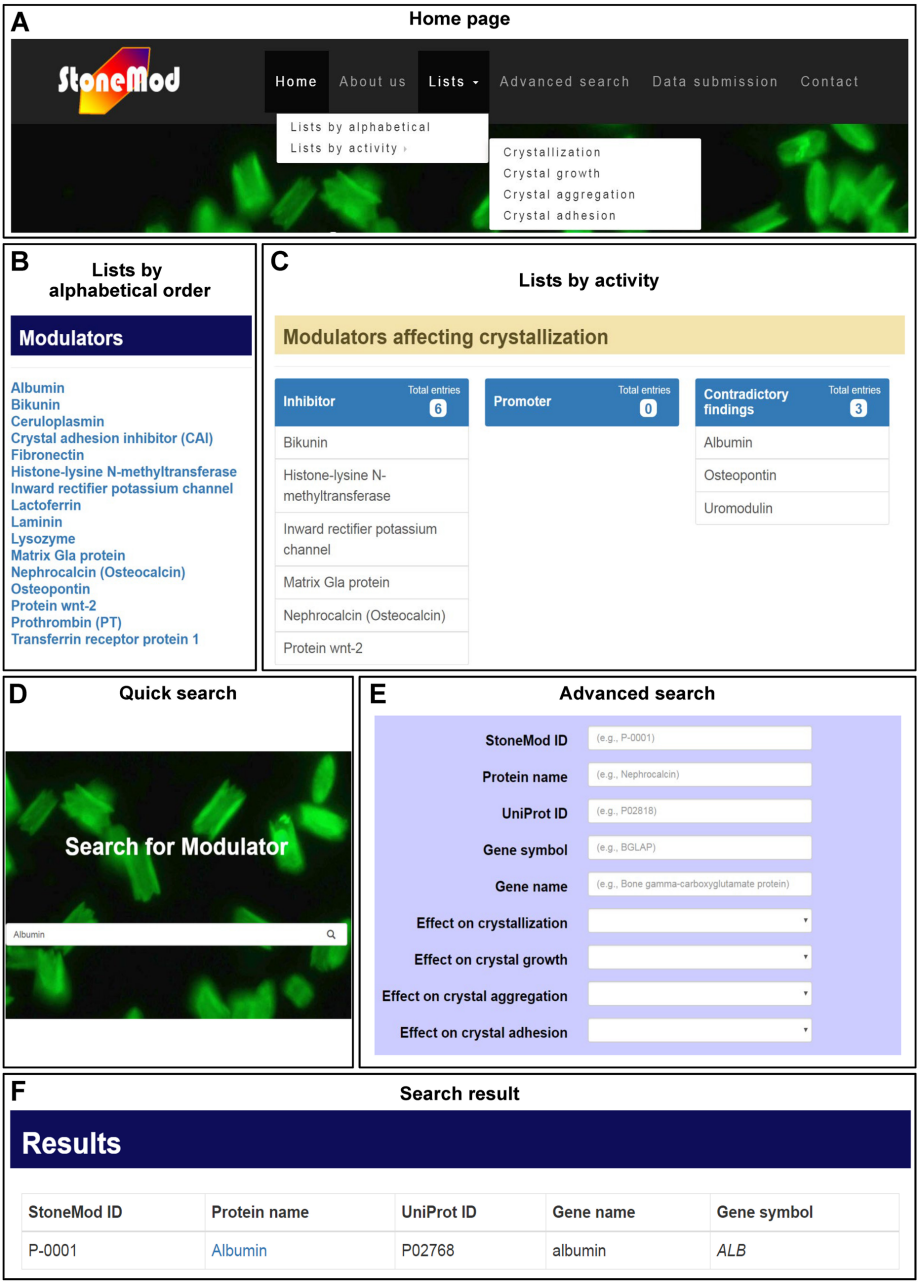
Presentation and appearance of the StoneMod database. (**A**): Home page with various tabbed document interfaces. (**B**): List by alphabetical order. (**C**): Lists by activity. (**D**): Quick search on the home page. (**E**): Advanced search using specified keyword(s). (**F**): Search result page for albumin.

The StoneMod database website is an open access resource for obtaining detailed information of kidney stone modulatory proteins that has been designed and organized for the ease of use and access. Using the MySQL schema as detailed in “Materials & Methods” (Fig. 3), the website tabbed document interfaces at this initial phase include “home”, “about us”, “lists”, “advanced search”, “data submission”, “contact”, and “help” tabs (Fig. 4A). The home page provides an overview of the database, brief background of kidney stone formation, and news of the database or related issues (Fig. 4A). This page also shows three most updated modulatory proteins and their modulatory activities.

**Figure 5:**
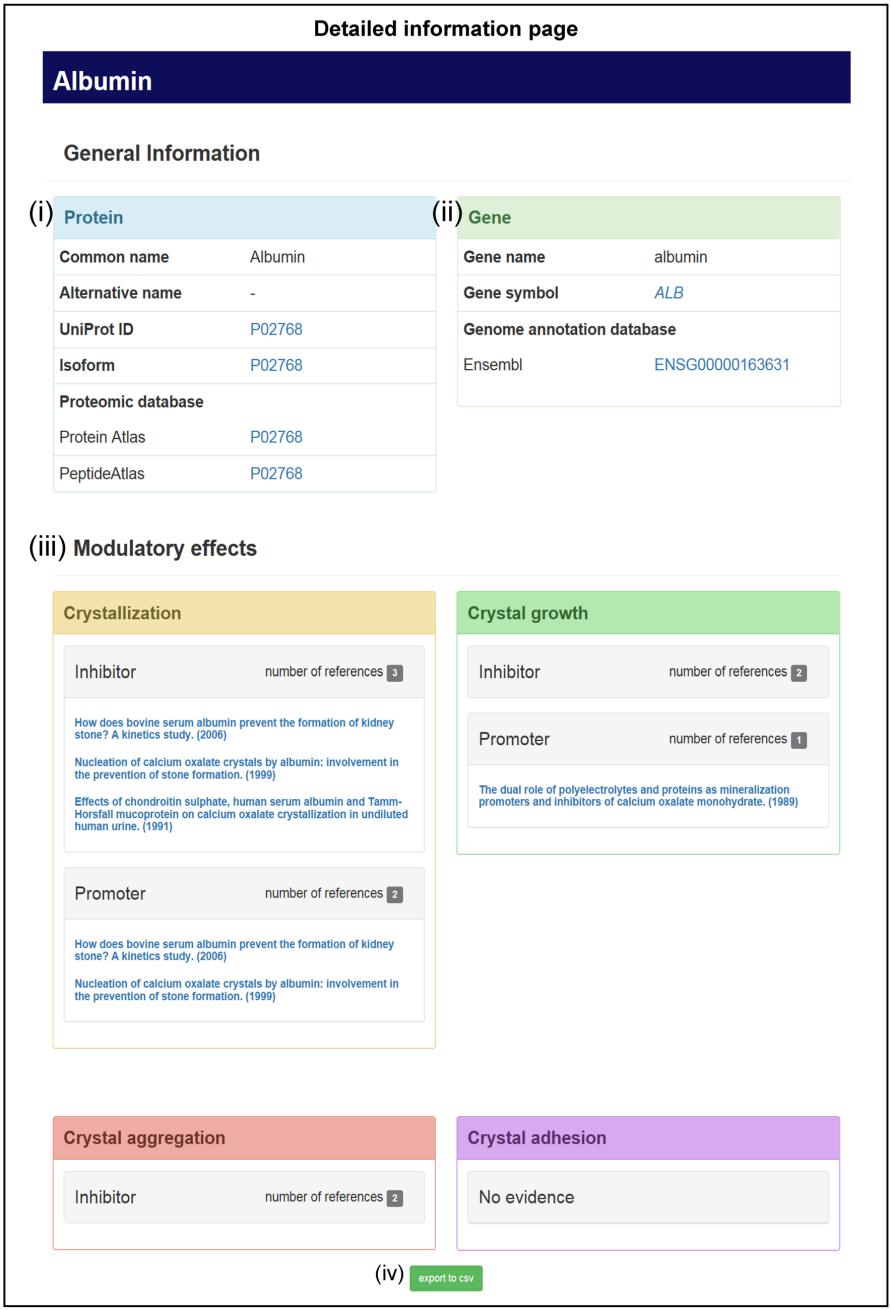
Detailed information page. (i): General information of protein. (ii): General information of gene. (iii): Modulatory effects. Moreover, the detailed information can be exported as .cvs file format (iv).

The lists menu provides two choices, in which modulators are sorted by alphabetical order or by activity involving crystallization, crystal growth, crystal aggregation, or crystal adhesion on renal tubular cells (Figs. 4B,C). For each step of kidney stone formation, individual modulatory proteins are categorized by their modulatory effects (e.g., promotion or inhibition). Quick search can be done through the home page using generalized keyword (e.g. protein common name, protein alternative name, gene name, gene symbol, UniProtKB accession number, etc.) (Fig. 4D). This allows the users to directly access the information of the protein or modulator of interest. In addition to the quick search, the users can perform advanced search by inputting specified and multiple search parameters (Fig. 4E). In either case, the search result will show brief information of the resulting modulator, including its StoneMod ID, protein name, UniProt ID, gene name, and gene symbol (Fig. 4F). Clicking the protein name will lead the users to the detailed information of each modulatory protein (Fig. 5).

### Relevant information of each modulatory protein

The detailed information page includes relevant data of each modulatory protein, including: (i) protein information; (ii) gene information; and (iii) modulatory effects (Table 2). The protein information (retrieved mainly from the UniProtKB database) includes protein common name, alternative name, UniProt ID, protein isoform (if any), and hyperlinks to the proteomic databases (i.e., Human Protein Atlas and PeptideAtlas) (Fig. 5; panel (i)). The gene information (retrieved mainly from the NCBI Gene database) provides gene name, gene symbol, and hyperlink to the gene annotation database (i.e., Ensembl) (Fig. 5; panel (ii)). Details of modulatory effects of each modulator (retrieved mainly from the PubMed search) include all relevant references of its promoting or inhibitory effect on crystallization, crystal growth, crystal aggregation, or crystal adhesion (Fig. 5; panel (iii)). Number of the references in each category is also summarized and shown on this page. Each reference is further linked to the PubMed literature resource. Finally, the StoneMod database also offers the users to download or export all the detailed information as comma-separated values (csv) file format by clicking “export to csv” icon at the bottom of the detailed information page (Fig. 5; panel (iv)).

**Table 2:** Details of the relevant information provided in the StoneMod database.

Type of the information	Items displayed	Description (with example)	Resource of the information
Protein information	Common name	Full name recommended by the UniProt consortium (e.g. Uromodulin)	UniProtKB^13^ (https://www.uniprot.org/)
	Alternative name	Synonym of the recommended full name (e.g. Tamm-Horsfall urinary glycoprotein)	UniProtKB^13^ (https://www.uniprot.org/)
	UniProt ID	The UniProtKB accession number of a protein that consists of 6 or 10 alphanumerical characters with a hyperlink to the UniProtKB website (e.g. P07911)	UniProtKB^13^ (https://www.uniprot.org/)
	Isoform	A hyperlink to the protein isoform (if any) in the UniProtKB website (e.g. P07911)	UniProtKB^13^ (https://www.uniprot.org/)
	Protein Atlas	The Protein Atlas identifier with a hyperlink to the Human Protein Atlas website (e.g. P07911)	Human Protein Atlas^14^ (https://www.proteinatlas.org/)
	PeptideAtlas	The PeptideAtlas identifier with a hyperlink to the PeptideAtlas website (e.g. P07911)	PeptideAtlas^15^ (https://www.peptideatlas.org/)
Gene information	Gene name	The official full name of the gene (e.g. uromodulin)	NCBI Gene^16^ (https://www.ncbi.nlm.nih.gov/gene)
	Gene symbol	The official symbol of the gene with a hyperlink to the NCBI Gene website (e.g. UMOD)	NCBI Gene^16^ (https://www.ncbi.nlm.nih.gov/gene)
	Ensembl	The Ensembl gene identifier in the Genome annotation database (e.g. ENSG00000169344)	Ensembl^18^ (https://www.ensembl.org/)
Modulatory effects	Crystallization	Modulatory effects on crystallization with references and hyperlinks to PubMed	PubMed searching with manual curation
	Crystal growth	Modulatory effects on crystal growth with references and hyperlinks to PubMed	PubMed searching with manual curation
	Crystal aggregation	Modulatory effects on crystal aggregation with references and hyperlinks to PubMed	PubMed searching with manual curation
	Crystal adhesion	Modulatory effects on crystal adhesion with references and hyperlinks to PubMed	PubMed searching with manual curation

Some of the modulators had contradictory results shown by different studies (mostly due to differential settings/parameters tested). They are then listed within “contradictory” category in the “lists by activity” tab. For example, there are three modulators (albumin, osteopontin, and uromodulin) that are in the “contradictory” category for crystal growth (Fig. 4C). The detailed information page of each protein will show all the contradictory data in one place (as in the case for albumin in Fig. 5; panel (iii), in which “modulatory effects” section shows all references for inhibitory and promoting effects of albumin on crystal growth.

### Data submission and update

In addition to periodic (monthly) deposition and update by our team, the StoneMod database also provides a submission form on “data submission” tab (Fig. 4A) to allow the users to directly deposit or update their own information into the database manually (note that the users must provide the PubMed ID or digital object identifier (DOI) of the published articles). After submission, each filled form will be directly sent to us for review. If the submitted references are relevant and show experimental evidence of modulatory effects of their proteins on kidney stone formation, they will be deposited and updated on the website within a week after submission. Finally, the latest deposited modulator will be highlighted on the home page and the submitter will be credited and notified.

## Conclusions

StoneMod is the first web-based database that provides relevant information of the kidney stone modulatory proteins with experimental evidence. The database has elements that are easy to use through the user-friendly web interface. Features of the StoneMod database enable the users to freely access to such information in one place. Moreover, the users can also submit their data to be deposited and updated. This database therefore will be a valuable resource of information for kidney stone research community.

## Methods

### Data collection and curation

Kidney stone modulatory data were collected and curated from published research articles with experimental evidence. Initially, all related articles were retrieved from PubMed database using the keywords: ""calcium oxalate"[All Fields] AND ("promote"[All Fields] OR "inhibit"[All Fields] OR "reduce"[All Fields] OR "modulate"[All Fields] OR "prevent"[All Fields] OR "enhance"[All Fields]) AND English[lang] *NOT Review[ptyp] NOT systematic[sb] NOT Comment[sb] NOT Editorial[ptyp]*" (Fig. 2). Thereafter, the data were manually filtered by including only mammalian proteins with experimental evidence of modulatory effects in kidney stone formation processes. For protein information, UniProtKB (https://www.uniprot.org/) was used to retrieve common name, alternative name, isoform, and UniProtKB ID^13^. Human Protein Atlas (https://www.proteinatlas.org/)^14^ and PeptideAtlas (https://www.peptideatlas.org/)^15^ were also used as the protein annotation database. For gene information, gene name and symbol were retrieved from the NCBI Gene database (https://www.ncbi.nlm.nih.gov/gene)^16^. Gene name and gene symbol are presented following the HUGO (Human Genome Organization) Gene Nomenclature guideline^17^. Gene annotation was retrieved from Ensembl database (https://www.ensembl.org/)^18^. Each modulatory protein was categorized by its effect (either promotion or inhibition) on kidney stone formation processes (i.e., crystallization, crystal growth, crystal aggregation, and crystal adhesion on renal tubular cells). Nevertheless, when the references showed inconclusive or contradictory data, the protein was classified into the contradictory category.

### Database implementation

StoneMod database website was built by using WampServer (https://www.wampserver.com), which is a freely open-source and cross-platform server that supports applications and creation of database using Apache2, PHP and MySQL in Linux subsystem. MySQL workbench (https://www.mysql.com) was chosen to manage the StoneMod database because of its ease of use. The MySQL schema used for the StoneMod database is illustrated in Fig. 3. The main relational database was structured with twelve tables of parameters, including protein, gene, isoform, crystallization, growth, aggregation, adhesion, effect, summary of crystallization, summary of growth, summary of aggregation, and summary of adhesion. Each table contained information represented in the column and data type, as well as links to the others through the relationship. PHP was also employed in combination with MySQL as the server-side script. In addition, the web framework development Bootstrap (https://getbootstrap.com/), which is the most popular framework for developing responsive website, and the JavaScript framework development JQuery (https://jquery.com/) were used for developing the web interface.

## Supplementary information

Supplementary information.
