# Supplementary material for: StoneMod: a database for kidney stone modulatory proteins with experimental evidence
Source: Sci Rep. 2020 Sep 15;10:15109. doi: 10.1038/s41598-020-71730-3 (PMC7493926; doi:10.1038/s41598-020-71730-3)
Supplement: Supplementary file 1 — Supplementary information. [file 41598_2020_71730_MOESM1_ESM.pdf]

SUPPORTING INFORMATION

StoneMod: A database for kidney stone modulatory proteins with experimental evidence

Supatcha Sassanarakkit, Paleerath Peerapen, and Visith Thongboonkerd (*correspondence to: vthongbo@yahoo.com)*

Supplementary Table S1: Details for sources of modulatory proteins, sample size, cells, animals and human subjects in each study included in the StoneMod database.

Study Title and References	PubMed ID	Sources of kidney stone modulatory proteins			
		Commercial	*In vitro*	Animal	Human
The calcium oxalate crystal growth inhibitor protein produced by mouse kidney cortical cells in culture is osteopontin.[1]	1414495	-	**Cellular secretome:** *Primary renal cortical tubular cells from C57b16 mice*	-	-
Effects of chondroitin sulphate, human serum albumin and Tamm-Horsfall mucoprotein on calcium oxalate crystallization in undiluted human urine.[2]	1909472	**Purified protein**	-	-	**Human urine:**<br>*No. of samples*: 11<br>*Gender:* Male<br>*Mean age:* 37 years<br>*Health status:* Healthy
Evidence that nephrocalcin and urine inhibit nucleation of calcium oxalate monohydrate crystals.[3]	1951713	-	-	-	**Human urine:**<br>*No. of samples*: 2<br>*Gender:* N/A<br>*Mean age:* N/A<br>*Health status:* 1 healthy and 1 stone former (recurrent calcium oxalate stone)
Inhibitors within the nephron.[4]	2008908	-	-	-	**Human urine:**<br>*No. of samples*: N/A<br>*Gender:* N/A<br>*Mean age:* N/A<br>*Health status:* Healthy and stone former
Does Tamm-Horsfall mucoprotein inhibit or promote calcium oxalate crystallization in human urine?[5]	2253402	-	-	-	**Human urine:**<br>*No. of samples*: 15<br>*Gender:* Male<br>*Mean age:* 30

					Health status: Healthy
The dual role of polyelectrolytes and proteins as mineralization promoters and inhibitors of calcium oxalate monohydrate.[6]	2476205	**Purified protein**	-	-	-
Possible role of Tamm-Horsfall glycoprotein in calcium oxalate crystallisation.[7]	2611614	-	-	-	**Human urine:**<br>*No. of samples*: 11<br>*Gender:* Male<br>*Mean age:* N/A<br>*Health status:* Healthy
Inhibition of calcium oxalate monohydrate crystal aggregation by urine proteins.[8]	2750929	**Purified protein**	-	-	**Human urine:**<br>*No. of samples*: N/A<br>*Gender:* N/A<br>*Mean age:* N/A (adult)<br>*Health status:* Healthy and stone former with recurrent calcium oxalate stone<br>**Calcium oxalate stone:**<br>*No. of samples*: N/A<br>*Gender:* N/A<br>*Mean age:* N/A (adult)<br>*Health status:* Stone former
Crystal adsorption and growth slowing by nephrocalcin, albumin, and Tamm-Horsfall protein.[9]	3202183	**Purified protein**	**Cellular secretome:**<br>*Human kidney cells*	-	**Human urine:**<br>*No. of samples*: N/A<br>*Gender:* N/A<br>*Mean age:* N/A (adult)<br>*Health status:* Healthy
Tamm-Horsfall mucoproteins promote calcium oxalate crystal formation in urine: quantitative studies.[10]	7057493	-	-	-	**Human urine:**<br>*No. of samples*: N/A<br>*Gender:* N/A<br>*Mean age:* N/A (adult)<br>*Health status:* Healthy
Tamm and Horsfall glycoprotein does not promote spontaneous precipitation and crystal growth of calcium oxalate *in vitro.*[11]	7086979	-	-	-	**Human urine:**<br>*No. of samples*: 15<br>*Gender:* N/A<br>*Mean age:* N/A<br>*Health status:* 6 healthy and 9

					stone formers with recurrent calcium stones
Characterization of uronic-acid-rich inhibitor of calcium oxalate crystallization isolated from rat urine.[12]	7676539	-	-	-	**Human urine:**<br>*No. of samples*: N/A<br>*Gender:* Male<br>*Mean age:* N/A (adult)<br>*Health status:* Healthy
Adhesion of calcium oxalate monohydrate crystals to renal epithelial cells is inhibited by specific anions.[13]	7733317	-	**Cellular secretome:**<br>*Human renal carcinoma cells*	-	**Human urine:**<br>*No. of samples*: 2<br>*Gender*: N/A<br>*Mean age*: N/A<br>*Health status*: Healthy
The role of Tamm-Horsfall mucoprotein in calcium oxalate crystallization. N-acetylcysteine--a new therapy for calcium oxalate urolithiasis.[14]	7953256	-	-	-	**Human urine:**<br>*No. of samples*: 16<br>*Gender:* N/A<br>*Mean age:* N/A<br>*Health status:* 10 healthy and 6 stone formers with recurrent calcium oxalate stones
Characterization of calcium-binding sites in the kidney stone inhibitor glycoprotein nephrocalcin with vanadyl ions: electron paramagnetic resonance and electron nuclear double resonance spectroscopy.[15]	7972057	-	-	**Animal kidney:**<br>*Bovine*	-
The effect of warfarin on urine calcium oxalate crystal growth inhibition and urinary excretion of calcium and nephrocalcin.[16]	8275352	-	-	-	**Human urine:**<br>*No. of samples*: N/A<br>*Gender:* N/A<br>*Mean age:* N/A<br>*Health status:* Healthy
Regulation of renal epithelial cell endocytosis of calcium oxalate monohydrate crystals.[17]	8498532	-	**Cellular secretome:**<br>*Human kidney cells*	-	**Human urine:**<br>*No. of samples*: 2<br>*Gender:* N/A<br>*Mean age:* N/A (adult)<br>*Health status:* Healthy

Effect of Tamm-Horsfall protein on calcium oxalate precipitation.[18]	8608191	-	-	-	**Human urine:**<br>*No. of samples*: N/A<br>*Gender:* Male and female<br>*Mean age:* N/A (adult)<br>*Health status:* Healthy
Identification of a macromolecular crystal growth inhibitor in human urine as osteopontin.[19]	8839390	-	-	-	**Human urine:**<br>*No. of samples*: N/A<br>*Gender:* N/A<br>*Mean age:* N/A<br>*Health status:* N/A
Characterization of Ca(2+)-binding sites in the kidney stone inhibitor glycoprotein nephrocalcin using vanadyl ions: different metal binding properties in strong and weak inhibitor proteins revealed by EPR and ENDOR.[20]	8942630	-	-	**Animal kidney:**<br>*Bovine*	-
Renal cell osteopontin production is stimulated by calcium oxalate monohydrate crystals.[21]	9067899	-	**Cellular secretome:**<br>*- Nontransformed African green monkey (BSC-1) cells*<br>*- Madin-Darby canine kidney (MDCK) cells*<br>*- BALB/c3T3 fibroblasts*	-	-
Contribution of human uropontin to inhibition of calcium oxalate crystallization.[22]	9453018	-	-	-	**Human urine:**<br>*No. of samples*: 13<br>*Gender:* 8 males, 5 females<br>*Mean age:* Males = 41, females = 27<br>*Health status:* Healthy
Identification of structural domains in inter-alpha-trypsin involved in calcium oxalate crystallization.[23]	9607205	-	-	-	**Human urine:**<br>*No. of samples*: N/A<br>*Gender:* N/A<br>*Mean age:* N/A<br>*Health status:* N/A<br>**Human serum:**<br>*No. of samples*: N/A<br>*Gender:* N/A<br>*Mean age:* N/A<br>*Health status:* N/A

Osteopontin antisense oligonucleotide inhibits adhesion of calcium oxalate crystals in Madin-Darby canine kidney cell.[24]	9751404	-	**Cellular secretome:** *Madin-Darby canine kidney (MDCK) cells*	-	-
Role of inter-alpha-inhibitor and its related proteins in urolithiasis. Purification of an inter-alpha-inhibitor related protein from the bovine kidney.[25]	10092154	-	-	**Animal kidney:** *Bovine*	-
Nucleation of calcium oxalate crystals by albumin: involvement in the prevention of stone formation.[26]	10231440	-	-	-	**Human serum:**<br>*No. of samples*: N/A<br>*Gender:* N/A<br>*Mean age:* N/A<br>*Health status:* N/A
Inhibition of calcium oxalate crystal growth and aggregation by prothrombin and its *fragments in vitro*: relationship between protein structure and inhibitory activity.[27]	10429186	-	-	-	**Human blood:**<br>*No. of samples*: N/A<br>*Gender:* N/A<br>*Mean age:* N/A<br>*Health status:* N/A
Role of urinary bikunin in the inhibition of calcium oxalate crystallization.[28]	10541269	-	-	-	**Human urine:**<br>*No. of samples*: N/A<br>*Gender:* N/A<br>*Mean age:* N/A<br>*Health status:* N/A
Prothrombin gene expression in rat kidneys provides an opportunity to examine its role in urinary stone pathogenesis.[29]	10541273	-	-	**Animal kidney:** *Wistar rats*	-
Citrate determines calcium oxalate crystallization kinetics and crystal morphology-studies in the presence of Tamm-Horsfall protein of a healthy subject and a severely recurrent calcium stone former.[30]	10692522	-	-	-	**Human urine:**<br>*No. of samples*: 2<br>*Gender:* Male<br>*Mean age:* 41<br>*Health status:* 1 healthy and 1 stone former with recurrent calcium stone
Fibronectin as a potent inhibitor of calcium oxalate urolithiasis.[31]	11025758	-	**Cellular secretome:** *Madin-Darby canine kidney (MDCK) cells*	**Animal kidney:** *8-week-old, specific pathogen-free, male Wistar rats*	**Human urine:**<br>*No. of samples*: 6<br>*Gender:* Male<br>*Mean age:* N/A (26-39 years)<br>*Health status:* Healthy

Expression of proteins that inhibit calcium oxalate crystallization *in vitro* in the urine of normal and stone-forming individuals.[32]	11136174	-	-	-	**Human urine:**<br>*No. of samples*: 85<br>*Gender:* 50 males, 35 females<br>*Mean age:* N/A<br>*Health status:* 24 healthy males, 19 healthy females, 26 male stone formers, 16 female stone formers
Strong inhibition of crystal-cell attachment by pediatric urinary macromolecules: a close relationship with high urinary citrate secretion.[33]	11549512	-	-	-	**Human urine:**<br>*No. of samples*: 21<br>*Gender:* Male<br>*Mean age:* children = 6.1, adults = 31.5<br>*Health status:* Healthy
Effect of prothrombin and its activation fragments on calcium oxalate crystal growth and aggregation in undiluted human urine *in vitro*: relationship between protein structure and inhibitory activity.[34]	11914105	-	-	-	**Human urine:**<br>*No. of samples*: 10<br>*Gender:* Male<br>*Mean age:* N/A<br>*Health status:* Healthy<br>**Human blood:**<br>*No. of samples*: N/A<br>*Gender:* N/A<br>*Mean age:* N/A<br>*Health status:* N/A
Role of Tamm-Horsfall protein and uromodulin in calcium oxalate crystallization.[35]	12424489	-	-	-	**Human urine:**<br>*No. of samples*: 30<br>*Gender:* 20 males, 10 females<br>*Mean age:* N/A<br>*Health status:* 10 healthy males, 5 healthy females and 5 pregnant females, 10 male stone formers
The role of osteopontin on calcium oxalate crystal formation.[36]	12706004	**Purified protein**	**Cellular secretome:**<br>*Engelbreth–Holm–Swarm mouse sarcoma cells*	**Animal serum:**<br>*Bovine*	**Human urine:**<br>*No. of samples*: N/A<br>*Gender:* N/A<br>*Mean age:* N/A<br>*Health status:* N/A<br>**Human milk:**<br>*No. of samples*: N/A<br>*Gender:* Female

					Mean age: N/A<br>*Health status:* N/A
Renal tubular cell injury and fibronectin.[37]	13680020	**Purified protein**	-	-	-
Identification of Bikunin isolated from human urine inhibits calcium oxalate crystal growth and its localization in the kidneys.[38]	14516400	-	-	-	**Human urine:**<br>*No. of samples*: 2<br>*Gender:* Male<br>*Mean age:* N/A<br>*Health status:* Healthy
Molecular modulation of calcium oxalate crystallization by osteopontin and citrate.[39]	14766970	-	-	-	**Human urine:**<br>*No. of samples*: N/A<br>*Gender:* N/A<br>*Mean age:* N/A<br>*Health status:* N/A
Renal epithelial cells constitutively produce a protein that blocks adhesion of crystals to their surface.[40]	15100100	-	**Cellular secretome:**<br>*Nontransformed African green monkey renal epithelial cells (BSC-1)*	-	-
Effects of urinary prothrombin fragment 1 in the formation of calcium oxalate calculus.[41]	15592049	-	-	-	**Human urine:**<br>*No. of samples*: 20<br>*Gender:* Male<br>*Mean age:* N/A (healthy = 28-43 years, stone former = 24-40 years)<br>*Health status:* 8 healthy, 12 calcium oxalate stone formers
Regulation by macromolecules of calcium oxalate crystal aggregation in stone formers.[42]	15864572	**Purified protein**	**Cellular secretome:**<br>*Mouse kidney cells*	**Animal milk:**<br>*Bovine*	**Human urine:**<br>*No. of samples*: 52<br>*Gender:* 38 males, 14 females<br>*Mean age:* Healthy = 46, stone former = 51<br>*Health status:* 20 healthy males, 5 healthy females, 18 male stone formers, 9 female stone formers

Urinary macromolecular inhibition of crystal adhesion to renal epithelial cells is impaired in male stone formers.[43]	16164655	-	-	-	**Human urine:**<br>*No. of samples*: 48<br>*Gender:* 34 males, 14 females<br>*Mean age:* Males = 56.7, females = 54.0<br>*Health status:* 17 healthy males, 7 healthy females,17 male stone formers, 7 female stone formers
Identification of human urinary trefoil factor 1 as a novel calcium oxalate crystal growth inhibitor.[44]	16308573	-	-	-	**Human urine:**<br>*No. of samples*: 53<br>*Gender:* 26 males, 27 females<br>*Mean age:* Healthy = 26.1, stone former = 45.3<br>*Health status:* 14 healthy males and 16 healthy females, 12 male stone formers, 11 female stone formers (18 new-onset calcium oxalate stone formers and 5 recurrent calcium oxalate stone formers)
How does bovine serum albumin prevent the formation of kidney stone? A kinetics study.[45]	16671718	**Purified protein**	-	-	-
Tamm-Horsfall protein in recurrent calcium kidney stone formers with positive family history: abnormalities in urinary excretion, molecular structure and function.[46]	17345077	-	-	-	**Human urine:**<br>*No. of samples*: 78<br>*Gender:* N/A<br>*Mean age:* N/A<br>*Health status:* 34 healthy, 44 recurrent calcium stone formers
Renal calcinosis and stone formation in mice lacking osteopontin, Tamm-Horsfall protein, or both.[47]	17898038	-	-	**Animal urine:**<br>*OPN-null mice*<br>*THP-null mice*	-
Control of calcium oxalate crystal growth by face-specific adsorption of an osteopontin phosphopeptide.[48]	17994739	**Synthesized phosphopeptide**	-	-	-

Urinary trefoil factor 1 is a novel potent inhibitor of calcium oxalate crystal growth and aggregation.[49]	18295252	-	-	-	**Human urine:**<br>*No. of samples*: 5<br>*Gender:* N/A<br>*Mean age:* N/A<br>*Health status:* Healthy
Phosphorylation of osteopontin is required for inhibition of calcium oxalate crystallization.[50]	18611047	**Synthesized phosphopeptide**	-	-	-
Phosphorylation of osteopontin peptides mediates adsorption to and incorporation into calcium oxalate crystals.[51]	18728346	**Synthesized phosphopeptide**	-	-	-
Kinetics of calcium oxalate crystal growth in the presence of osteopontin isoforms: an analysis by scanning confocal interference microcopy.[52]	19189038	**Purified protein**	-	**Animal bone:**<br>*Rat*<br>**Animal milk:**<br>*Cow*	-
Modulation of calcium oxalate dihydrate growth by selective crystal-face binding of phosphorylated osteopontin and polyaspartate peptide showing occlusion by sectoral (compositional) zoning.[53]	19581305	-	-	-	**Human urine:**<br>*No. of samples*: 6<br>*Gender:* Male<br>*Mean age:* N/A (23-45 years)<br>*Health status:* Healthy<br>**Stone:**<br>*No. of samples*: 2<br>*Gender:* Female<br>*Mean age:* 67.5<br>*Health status:* Stone former
The effect of intracrystalline and surface-bound osteopontin on the attachment of calcium oxalate dihydrate crystals to Madin-Darby canine kidney (MDCK) cells in ultrafiltered human urine.[54]	21883862	-	-	-	**Human urine:**<br>*No. of samples*: 5<br>*Gender:* 2 males, 3 females<br>*Mean age:* N/A<br>*Health status:* Healthy<br>**Human milk:**<br>*No. of samples*: N/A<br>*Gender:* Female<br>*Mean age:* N/A<br>*Health status:* N/A
On the catalysis of calcium oxalate dihydrate formation by osteopontin peptides.[55]	22503630	**Synthesized phosphopeptide**	-	-	-

Novel antilithiatic cationic proteins from human calcium oxalate renal stone matrix identified by MALDI-TOF-MS endowed with cytoprotective potential: an insight into the molecular mechanism of urolithiasis[56]	23123287	-	-	-	**Human stone:**<br>*No. of samples*: N/A<br>*Gender:* N/A<br>*Mean age:* N/A (>25 years)<br>*Health status:* Stone former
Reversible inhibition of calcium oxalate monohydrate growth by an osteopontin phosphopeptide.[57]	23611580	**Synthesized phosphopeptide**	-	-	-
Peptides of Matrix Gla protein inhibit nucleation and growth of hydroxyapatite and calcium oxalate monohydrate crystals.[58]	24265810	**Synthesized phosphopeptide**	-	-	-
Natural promoters of calcium oxalate monohydrate crystallization.[59]	25119124	**Purified protein**	-	-	-
The Long Pentraxin PTX3 Is an Endogenous Inhibitor of Hyperoxaluria-Related Nephrocalcinosis and Chronic Kidney Disease.[60]	30319631	-	**Cellular secretome:**<br>*PerC6 human cell line*	-	-
SIRT3 inhibited the formation of calcium oxalate-induced kidney stones through regulating NRF2/HO-1 signaling pathway.[61]	30548662	-	**Intracellular protein:**<br>*HK-2 cells*	-	-
Modulatory effects of fibronectin on calcium oxalate crystallization, growth, aggregation, adhesion on renal tubular cells, and invasion through extracellular matrix.[62]	30701361	**Purified protein**		-	-

N/A = not available or not reported.

REFERENCES

1 Worcester, E. M., Blumenthal, S. S., Beshensky, A. M. & Lewand, D. L. The calcium oxalate crystal growth inhibitor protein produced by mouse kidney cortical cells in culture is osteopontin. *J Bone Miner Res* **7**, 1029-1036, doi:10.1002/jbmr.5650070905 (1992).

2 Ryall, R. L., Harnett, R. M., Hibberd, C. M., Edyvane, K. A. & Marshall, V. R. Effects of chondroitin sulphate, human serum albumin and Tamm-Horsfall mucoprotein on calcium oxalate crystallization in undiluted human urine. *Urol Res* **19**, 181-188, doi:10.1007/BF00303747 (1991).

3 Asplin, J., DeGanello, S., Nakagawa, Y. N. & Coe, F. L. Evidence that nephrocalcin and urine inhibit nucleation of calcium oxalate monohydrate crystals. *Am J Physiol* **261**, F824-830, doi:10.1152/ajprenal.1991.261.5.F824 (1991).

4 Coe, F. L., Nakagawa, Y. & Parks, J. H. Inhibitors within the nephron. *Am. J Kidney Dis* **17**, 407-413 (1991).

5 Grover, P. K., Ryall, R. L. & Marshall, V. R. Does Tamm-Horsfall mucoprotein inhibit or promote calcium oxalate crystallization in human urine? *Clin. Chim. Acta* **190**, 223-238 (1990).

6 Campbell, A. A., Ebrahimpour, A., Perez, L., Smesko, S. A. & Nancollas, G. H. The dual role of polyelectrolytes and proteins as mineralization promoters and inhibitors of calcium oxalate monohydrate. *Calcif Tissue Int* **45**, 122-128, doi:10.1007/BF02561411 (1989).

7 Yoshioka, T. *et al.* Possible role of Tamm-Horsfall glycoprotein in calcium oxalate crystallisation. *Br J Urol* **64**, 463-467, doi:10.1111/j.1464-410x.1989.tb05277.x (1989).

8 Hess, B., Nakagawa, Y. & Coe, F. L. Inhibition of calcium oxalate monohydrate crystal aggregation by urine proteins. *Am. J Physiol* **257**, F99-106 (1989).

9 Worcester, E. M., Nakagawa, Y., Wabner, C. L., Kumar, S. & Coe, F. L. Crystal adsorption and growth slowing by nephrocalcin, albumin, and Tamm-Horsfall protein. *Am. J Physiol* **255**, F1197-F1205 (1988).

10 Rose, G. A. & Sulaiman, S. Tamm-Horsfall mucoproteins promote calcium oxalate crystal formation in urine: quantitative studies. *J Urol* **127**, 177-179, doi:10.1016/s0022-5347(17)53656-3 (1982).

11 Kitamura, T. & Pak, C. Y. Tamm and Horsfall glycoprotein does not promote spontaneous precipitation and crystal growth of calcium oxalate in vitro. *J Urol* **127**, 1024-1026, doi:10.1016/s0022-5347(17)54180-4 (1982).

12 Atmani, F. & Khan, S. R. Characterization of uronic-acid-rich inhibitor of calcium oxalate crystallization isolated from rat urine. *Urol Res* **23**, 95-101, doi:10.1007/BF00307939 (1995).

13 Lieske, J. C., Leonard, R. & Toback, F. G. Adhesion of calcium oxalate monohydrate crystals to renal epithelial cells is inhibited by specific anions. *Am. J Physiol* **268**, F604-F612 (1995).

14 Fan, J. & Shen, S. J. The role of Tamm-Horsfall mucoprotein in calcium oxalate crystallization. N-acetylcysteine--a new therapy for calcium oxalate urolithiasis. *Br J Urol* **74**, 288-293, doi:10.1111/j.1464-410x.1994.tb16612.x (1994).

15 Mustafi, D. & Nakagawa, Y. Characterization of calcium-binding sites in the kidney stone inhibitor glycoprotein nephrocalcin with vanadyl ions: electron paramagnetic resonance and electron nuclear double resonance spectroscopy. *Proc Natl Acad Sci U S A* **91**, 11323-11327, doi:10.1073/pnas.91.24.11323 (1994).

16 Worcester, E. M., Sebastian, J. L., Hiatt, J. G., Beshensky, A. M. & Sadowski, J. A. The effect of warfarin on urine calcium oxalate crystal growth inhibition and urinary excretion of calcium and nephrocalcin. *Calcif Tissue Int* **53**, 242-248, doi:10.1007/BF01320909 (1993).

17 Lieske, J. C. & Toback, F. G. Regulation of renal epithelial cell endocytosis of calcium oxalate monohydrate crystals. *Am. J Physiol* **264**, F800-F807 (1993).

18 Benkovic, J., Furedi-Milhofer, H., Hlady, V., Cvoriscec, D. & Stavljenic-Rukavina, A. Effect of Tamm-Horsfall protein on calcium oxalate precipitation. *Eur. J Clin. Chem. Clin. Biochem* **33**, 705-710 (1995).

19 Sorensen, S., Justesen, S. J. & Johnsen, A. H. Identification of a macromolecular crystal growth inhibitor in human urine as osteopontin. *Urol. Res* **23**, 327-334 (1995).

20 Mustafi, D. & Nakagawa, Y. Characterization of Ca(2+)-binding sites in the kidney stone inhibitor glycoprotein nephrocalcin using vanadyl ions: different metal binding properties in strong and weak inhibitor proteins revealed by EPR and ENDOR. *Biochemistry* **35**, 14703-14709, doi:10.1021/bi961200j (1996).

21 Lieske, J. C., Hammes, M. S., Hoyer, J. R. & Toback, F. G. Renal cell osteopontin production is stimulated by calcium oxalate monohydrate crystals. *Kidney Int* **51**, 679-686, doi:10.1038/ki.1997.98 (1997).

22 Asplin, J. R., Arsenault, D., Parks, J. H., Coe, F. L. & Hoyer, J. R. Contribution of human uropontin to inhibition of calcium oxalate crystallization. *Kidney Int* **53**, 194-199, doi:10.1046/j.1523-1755.1998.00739.x (1998).

23 Kobayashi, H., Shibata, K., Fujie, M., Sugino, D. & Terao, T. Identification of structural domains in inter-alpha-trypsin involved in calcium oxalate crystallization. *Kidney Int* **53**, 1727-1735 (1998).

24 Yamate, T., Kohri, K., Umekawa, T., Iguchi, M. & Kurita, T. Osteopontin antisense oligonucleotide inhibits adhesion of calcium oxalate crystals in Madin-Darby canine kidney cell. *J Urol* **160**, 1506-1512 (1998).

25 Atmani, F. & Khan, S. R. Role of inter-alpha-inhibitor and its related proteins in urolithiasis. Purification of an inter-alpha-inhibitor related protein from the bovine kidney. *Urol. Res* **27**, 57-61 (1999).

26 Cerini, C. *et al.* Nucleation of calcium oxalate crystals by albumin: involvement in the prevention of stone formation. *Kidney Int* **55**, 1776-1786, doi:10.1046/j.1523-1755.1999.00426.x (1999).

27 Grover, P. K. & Ryall, R. L. Inhibition of calcium oxalate crystal growth and aggregation by prothrombin and its fragments in vitro: relationship between protein structure and inhibitory activity. *Eur. J Biochem* **263**, 50-56 (1999).

28 Atmani, F. & Khan, S. R. Role of urinary bikunin in the inhibition of calcium oxalate crystallization. *J Am. Soc. Nephrol* **10 Suppl 14**, S385-S388 (1999).

29 Grover, P. K., Stapleton, A. M. & Ryall, R. L. Prothrombin gene expression in rat kidneys provides an opportunity to examine its role in urinary stone pathogenesis. *J Am Soc Nephrol* **10 Suppl 14**, S404-407 (1999).

30 Hess, B., Jordi, S., Zipperle, L., Ettinger, E. & Giovanoli, R. Citrate determines calcium oxalate crystallization kinetics and crystal morphology-studies in the presence of Tamm-Horsfall protein of a healthy subject and a severely recurrent calcium stone former. *Nephrol. Dial. Transplant* **15**, 366-374 (2000).

31 Tsujihata, M. *et al.* Fibronectin as a potent inhibitor of calcium oxalate urolithiasis. *J Urol* **164**, 1718-1723 (2000).

32 Hedgepeth, R. C., Yang, L., Resnick, M. I. & Marengo, S. R. Expression of proteins that inhibit calcium oxalate crystallization in vitro in the urine of normal and stone-forming individuals. *Am. J Kidney Dis* **37**, 104-112 (2001).

33 Miyake, O. *et al.* Strong inhibition of crystal-cell attachment by pediatric urinary macromolecules: a close relationship with high urinary citrate secretion. *Urology* **58**, 493-497, doi:10.1016/s0090-4295(01)01257-2 (2001).

34 Grover, P. K. & Ryall, R. L. Effect of prothrombin and its activation fragments on calcium oxalate crystal growth and aggregation in undiluted human urine in vitro: relationship between protein structure and inhibitory activity. *Clin Sci (Lond)* **102**, 425-434 (2002).

35 Carvalho, M., Mulinari, R. A. & Nakagawa, Y. Role of Tamm-Horsfall protein and uromodulin in calcium oxalate crystallization. *Braz J Med Biol Res* **35**, 1165-1172, doi:10.1590/s0100-879x2002001000009 (2002).

36 Konya, E., Umekawa, T., Iguchi, M. & Kurita, T. The role of osteopontin on calcium oxalate crystal formation. *Eur Urol* **43**, 564-571, doi:10.1016/s0302-2838(03)00088-5 (2003).

37 Tsujihata, M. *et al.* Renal tubular cell injury and fibronectin. *Urol. Res* **31**, 368-373 (2003).

38 Okuyama, M., Yamaguchi, S. & Yachiku, S. Identification of bikunin isolated from human urine inhibits calcium oxalate crystal growth and its localization in the kidneys. *Int J Urol* **10**, 530-535, doi:10.1046/j.1442-2042.2003.00677.x (2003).

39 Qiu, S. R. *et al.* Molecular modulation of calcium oxalate crystallization by osteopontin and citrate. *Proc Natl Acad Sci U S A* **101**, 1811-1815, doi:10.1073/pnas.0307900100 (2004).

40 Kumar, V., Yu, S., Farell, G., Toback, F. G. & Lieske, J. C. Renal epithelial cells constitutively produce a protein that blocks adhesion of crystals to their surface. *Am. J Physiol Renal Physiol* **287**, F373-F383 (2004).

41 Liu, J., Chen, J., Wang, T., Wang, S. & Ye, Z. Effects of urinary prothrombin fragment 1 in the formation of calcium oxalate calculus. *J Urol* **173**, 113-116, doi:10.1097/01.ju.0000146847.24571.c8 (2005).

42 Wesson, J. A., Ganne, V., Beshensky, A. M. & Kleinman, J. G. Regulation by macromolecules of calcium oxalate crystal aggregation in stone formers. *Urol. Res* **33**, 206-212 (2005).

43 Kumar, V., Pena de la Vega, L., Farell, G. & Lieske, J. C. Urinary macromolecular inhibition of crystal adhesion to renal epithelial cells is impaired in male stone formers. *Kidney Int* **68**, 1784-1792, doi:10.1111/j.1523-1755.2005.00595.x (2005).

44 Chutipongtanate, S. *et al.* Identification of human urinary trefoil factor 1 as a novel calcium oxalate crystal growth inhibitor. *J Clin. Invest* **115**, 3613-3622 (2005).

45 Liu, J., Jiang, H. & Liu, X. Y. How does bovine serum albumin prevent the formation of kidney stone? A kinetics study. *J Phys Chem B* **110**, 9085-9089, doi:10.1021/jp057403b (2006).

46 Jaggi, M., Nakagawa, Y., Zipperle, L. & Hess, B. Tamm-Horsfall protein in recurrent calcium kidney stone formers with positive family history: abnormalities in urinary excretion, molecular structure and function. *Urol Res* **35**, 55-62, doi:10.1007/s00240-007-0083-7 (2007).

47 Mo, L. *et al.* Renal calcinosis and stone formation in mice lacking osteopontin, Tamm-Horsfall protein, or both. *Am. J Physiol Renal Physiol* **293**, F1935-F1943 (2007).

48 Grohe, B. *et al.* Control of calcium oxalate crystal growth by face-specific adsorption of an osteopontin phosphopeptide. *J Am Chem Soc* **129**, 14946-14951, doi:10.1021/ja0745613 (2007).

49 Thongboonkerd, V., Chutipongtanate, S., Semangoen, T. & Malasit, P. Urinary trefoil factor 1 is a novel potent inhibitor of calcium oxalate crystal growth and aggregation. *J Urol* **179**, 1615-1619 (2008).

50 Wang, L. *et al.* Phosphorylation of osteopontin is required for inhibition of calcium oxalate crystallization. *J Phys. Chem. B* **112**, 9151-9157 (2008).

51 O'Young, J. *et al.* Phosphorylation of osteopontin peptides mediates adsorption to and incorporation into calcium oxalate crystals. *Cells Tissues Organs* **189**, 51-55, doi:10.1159/000151724 (2009).

52 Langdon, A. *et al.* Kinetics of calcium oxalate crystal growth in the presence of osteopontin isoforms: an analysis by scanning confocal interference microcopy. *Calcif Tissue Int* **84**, 240-248, doi:10.1007/s00223-008-9215-5 (2009).

53 Chien, Y. C. *et al.* Modulation of calcium oxalate dihydrate growth by selective crystal-face binding of phosphorylated osteopontin and polyaspartate peptide showing occlusion by sectoral (compositional) zoning. *J Biol Chem* **284**, 23491-23501, doi:10.1074/jbc.M109.021899 (2009).

54 Thurgood, L. A., Sorensen, E. S. & Ryall, R. L. The effect of intracrystalline and surface-bound osteopontin on the attachment of calcium oxalate dihydrate crystals to Madin-Darby canine kidney (MDCK) cells in ultrafiltered human urine. *BJU Int* **109**, 1100-1109, doi:10.1111/j.1464-410X.2011.10530.x (2012).

55 Chan, B. P. *et al.* On the catalysis of calcium oxalate dihydrate formation by osteopontin peptides. *Colloids Surf B Biointerfaces* **96**, 22-28, doi:10.1016/j.colsurfb.2012.03.015 (2012).

56 Aggarwal, K. P., Tandon, S., Naik, P. K., Singh, S. K. & Tandon, C. Novel antilithiatic cationic proteins from human calcium oxalate renal stone matrix identified by MALDI-TOF-MS endowed with cytoprotective potential: an insight into the molecular mechanism of urolithiasis. *Clin Chim Acta* **415**, 181-190, doi:10.1016/j.cca.2012.10.040 (2013).

57 Nene, S. S., Hunter, G. K., Goldberg, H. A. & Hutter, J. L. Reversible inhibition of calcium oxalate monohydrate growth by an osteopontin phosphopeptide. *Langmuir* **29**, 6287-6295, doi:10.1021/la400891b (2013).

58 Goiko, M. *et al.* Peptides of Matrix Gla protein inhibit nucleation and growth of hydroxyapatite and calcium oxalate monohydrate crystals. *PLoS One* **8**, e80344, doi:10.1371/journal.pone.0080344 (2013).

59 Farmanesh, S. *et al.* Natural promoters of calcium oxalate monohydrate crystallization. *J Am. Chem. Soc* **136**, 12648-12657 (2014).

60 Marschner, J. A. *et al.* The Long Pentraxin PTX3 Is an Endogenous Inhibitor of Hyperoxaluria-Related Nephrocalcinosis and Chronic Kidney Disease. *Front Immunol* **9**, 2173, doi:10.3389/fimmu.2018.02173 (2018).

61 Xi, J. *et al.* SIRT3 inhibited the formation of calcium oxalate-induced kidney stones through regulating NRF2/HO-1 signaling pathway. *J Cell Biochem*, doi:10.1002/jcb.28109 (2018).

62 Khamchun, S., Sueksakit, K., Chaiyarit, S. & Thongboonkerd, V. Modulatory effects of fibronectin on calcium oxalate crystallization, growth, aggregation, adhesion on renal tubular cells, and invasion through extracellular matrix. *J Biol. Inorg. Chem* **24**, 235-246 (2019).
